# Motivating Pulse-Centric Eating Patterns to Benefit Human and Environmental Well-Being

**DOI:** 10.3390/nu12113500

**Published:** 2020-11-14

**Authors:** Chelsea Didinger, Henry Thompson

**Affiliations:** 1 Department of Food Science and Human Nutrition, Colorado State University, Fort Collins, CO 80523, USA; chelsea.didinger@colostate.edu; 2 Cancer Prevention Laboratory, Colorado State University, Fort Collins, CO 80523, USA

**Keywords:** pulses, legumes, microbiome, obesity, sustainability, chronic disease prevention, food security, behavior change, motivation

## Abstract

Pulses (e.g., lentil, common bean, chickpea, and dry pea) are linked to a myriad of positive human and environmental health impacts, making them an ideal food for wise and conscientious global citizens. In addition, pulses are affordable and shelf-stable. The combination of these factors, an elevated consumer interest in plant-based diets, and the COVID-19 pandemic resulted in increased purchasing of pulses and even empty grocery store shelves. Although pulses have many associated benefits, some consumers are hesitant to regularly eat pulses, claiming concerns of abdominal discomfort or a lack of knowledge on how to best prepare pulses. To capitalize on increased consumer interest and purchasing of pulses, now is the time for outreach efforts that address these concerns and the positive outcomes associated with pulses, thereby promoting public and environmental health. Consumers must actively decide to add pulses to their grocery lists and incorporate them into their regular eating patterns. Motivation to adopt new eating habits is essential because knowledge alone does not result in behavior change. Thus, to mitigate perceived barriers and drive consumption, we suggest application of the Information-Motivation-Behavioral Skills Model and emphasis of three main benefits of pulses as motivators: (1) culinary versatility, (2) sustainability, and (3) healthfulness.

## 1. Introduction

Pulses (e.g., lentil, common bean, chickpea, and dry pea), the dry, edible seeds of grain legumes, are widely recognized for their potential to simultaneously promote human and environmental health [1,2]. Domesticated around 8000–10,000 years ago [3], pulses are a traditional staple food across the globe. Despite the numerous benefits associated with growing and eating pulses, consumption in recent decades has precipitously declined, especially in developed countries [4]. Several perceived barriers may contribute to consumer hesitation to eat more pulses, including concern over anti-nutrients, long cooking times, lack of knowledge of how to prepare pulses, and the perception that pulses are “poor person’s food” [5,6]. There is strong support in the scientific literature to address these concerns, which could help reverse the trend of declining consumption.

Purchases during the COVID-19 pandemic dispelled the myth that consumers will not actively seek out and buy pulses. Instead, demand for pulses increased [7] so much that it left many grocery store shelves empty or minimally supplied. This indicates that consumers already had the underlying knowledge that pulses are shelf-stable, economical, and healthful; however, they may have lacked the motivation to change their buying and eating habits prior to the food system disruptions caused by the COVID-19 pandemic. With consumer pantries better stocked with pulses, now is the time to establish and routinize pulse-rich eating patterns, thereby promoting public health.

## 2. Reasons to Eat Pulses

Although nutrition knowledge is essential to healthy food behaviors, knowledge alone does not appear to be sufficient to induce food choices [8]. To promote health behaviors, such as regularly eating pulses, the Information-Motivation-Behavioral Skills (IMB) Model [9] provides insights. To increase consumption, it is important to have a combination of information about the benefits of pulses, motivation to eat them, and the behavioral skills to prepare them. Two major components of motivation—personal and social motivation—are influenced by personal attitudes, cultural norms, and social support. Recently, well-known cooking websites (e.g., New York Times Cooking, Bon Appetit) have released many recipes highlighting pulses as well as other pantry staples. Similarly, organizations like the United States Department of Agriculture (USDA) and Extension provide outreach materials on cooking with pulses. Such outlets can help provide the social motivation and behavioral skills to cook with pulses.

**Figure 1:**
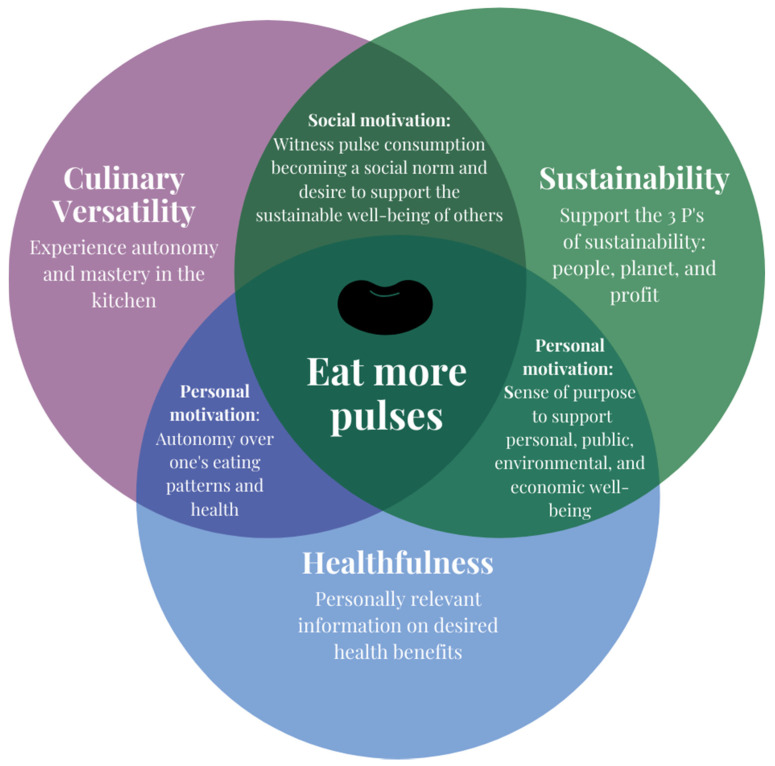
Emphasizing the culinary versatility, sustainability, and healthfulness of pulses to drive social and personal motivation to increase consumption.

To motivate individuals on a personal level, we suggest considering consumer autonomy (i.e., the need to direct one’s own actions), mastery (i.e., the desire to improve), and purpose (i.e., being motivated by a larger goal) [10]. The countless creative applications of pulses in the kitchen can inspire autonomy; as consumers master preparation skills, they will want to further experiment with different uses of pulses; and the benefits to personal, public, and environmental health provide a purpose to eat pulses. Thus, to motivate consumers to reap all the benefits pulses have to offer, we suggest highlighting three key aspects: their culinary versatility, sustainability, and healthfulness (Figure 1).

### 2.1. Culinary Versatility

For regular consumption to be feasible, it is essential that people enjoy eating pulses and can easily and habitually include them in daily meals. Doma and colleagues (2019) found that a main barrier to consumption was that because pulses such as common bean were not part of the traditional diets of participants, they did not think to include them in meals [6]. Pulses have been a staple food since ancient times; thus, there is a rich repertoire of pulse-based recipes worldwide from which to draw upon for inspiration, such as dal, falafel, pasta e ceci, hummus, bean paste desserts, and stewed beans. Even beyond the most commonly consumed pulses (e.g., chickpeas, common beans, dry peas, and lentils), there are other pulses (e.g., mung beans, lupins, and cowpeas) that provide additional exciting culinary and nutritional opportunities.

The American Pulse Association calls pulses the world’s most versatile superfood [11]. Indeed, pulses are highly adaptable. They can be used as powders or purees to significantly boost nutritional value without dramatically altering the flavor, work well in sweet and savory cooking and baking, are easily added to a wide range of dishes (e.g., smoothies, salads, pastas, dips, and stews), and can be applied in healthful substitutions (e.g., as a substitute for cream or cheese in sauces). The variety of pulse types available coupled with canned pulses, pre-cooked pulses in the refrigerated section, and choices of a multitude of pulse-based products also makes enjoying pulses feasible for people with all levels of culinary skills, from beginner to advanced.

Encouraging awareness of the many different pulses can allow consumers to take advantage of this diversity. For instance, lentils can be prepared in approximately the same amount of time as brown rice, thereby helping consumers to quickly incorporate more pulses into meals. Utilizing such knowledge is one way to address long cooking times, a perceived barrier to pulse consumption. Furthermore, although some may think of including pulses only during the dinner meal [6], there are innumerable ways to incorporate them into all meals and a variety of snacks.

We can introduce consumers to the versatility of pulses and the potential to include them during any mealtime through existing resources from the American Pulse Association and USA Dry Pea and Lentil Council (see pulses.org), which allows users to search recipe banks based on features like meal type (e.g., breakfast, snack, or dessert) and pulse type, as well as providing information on cooking tips, simple weekly meal plans, and more. Extension can also play a role in promoting awareness of pulses through hands-on classes and distribution of outreach materials that showcase the underappreciated versatility of pulses. Preparation and time-saving tips can also be taught through these materials, such as how soaking pulses in a salt solution significantly shortens cooking time [12] and how large batches can be cooked and frozen for later use because pulses freeze well. Recipes for all mealtimes and simple ‘cooking hacks’ like these helps make increasing consumption more feasible and exciting.

In addition to more traditional recipes and the roles pulses play in their whole form, there are nearly endless uses for pulses in the food industry. Pulses demonstrate numerous beneficial properties, such as gelation and emulsification, and pulse powders have functional properties and nutrient enrichment applications [2,13]. For example, bean flour can increase the amount of beneficial phenolic compounds in spaghetti [14] and levels of fiber, protein, and antioxidants in snack bars [15]. Also, pulses are a naturally gluten-free, high-protein, and high-fiber alternative to wheat flour in products such as baked and extruded goods [16]. Pulse powders, also referred to as flours, are becoming increasingly available in the market for consumers to use in the home in both savory and sweet recipes, like fritters and muffins. Importantly, innovative new products made with pulse powders (e.g., protein powders, black bean pasta, chickpea orzo, crackers, chips, and breakfast cereals) provide consumers the opportunity to effortlessly include pulses while still enjoying more familiar dishes, thus making adoption of a pulse-centric eating pattern feel more fluid and not like a difficult behavior change. Government data on foods and meal components most commonly eaten by consumers can be utilized to drive development of new pulse-based products designed to meet consumer taste and demands, such as pasta noodles and cream sauce substitutions. Moreover, exciting new technologies like machine learning and artificial intelligence may help predict consumer product preferences and generate meal plans targeted to consumer taste [17,18,19].

Ultimately, the public is more likely to increase consumption if doing so is convenient, delicious, nutritious, and affordable [20]. Canned pulses and new pulse-based products offer quick and tasty options. Furthermore, pulses are known for their affordability [21], differentiating them from other ‘superfoods’ that may be difficult to procure and expensive. Pulses can be utilized as the satiating base of a meal, to stretch meals (e.g., blend with meat as a cost-effective meat extender in dishes like lasagna), or to make dishes like salads more satisfying. At an economical price of less than the average candy bar, a can of pulses fosters autonomy in the kitchen and has excellent value for consumers because it has innumerable applications and is health-promoting.

### 2.2. Sustainability

To better motivate increased production and consumption, it is important to not only consider environmental impacts, but to examine sustainability in a more nuanced manner. Pulses directly support all three P’s of sustainability: planet, people, and profit [22]. This unique ability to simultaneously advance environmental goals along with the social, physical, and economic well-being of people around the world should be highlighted.

Pulses provide diverse agricultural and ecosystem services, such as weed suppression, increasing the yield of subsequent or associated crops, and improving soil fertility [23,24]. Well-known for their ability to fix nitrogen in the soil, pulses can also enrich soils through their utilization as green and brown manures, potentially reducing fossil-based nitrogen fertilizer inputs and benefitting future crops [25]. Water scarcity is another threat to food systems and grower livelihoods. However, pulses demonstrate high water use efficiency due to features such as their deep root system [24]. Thus, diversifying cropping systems with pulses can conserve soil water and reduce irrigation requirements and costs [26]. Pulses also use significantly less water than oilseed legumes: producing one pound of pulses only takes 43 gallons of water, whereas it takes 216 and 368 gallons to produce one pound of soybeans and peanuts, respectively [27]. Certain pulse cultivars are known for drought hardiness and climate resiliency, such as black-eyed peas and horsegram [28,29,30]. Thus, pulses are a sustainable and viable option to generate profits and improve food security even on marginal lands.

Pulses can also play a substantial role in climate change mitigation. If consumers shift to eating patterns rich in plant-based, pulse protein, this can greatly reduce greenhouse gas emissions. Harwatt and colleagues (2017) demonstrated that substituting beans for beef could achieve 46–74% of the reductions of greenhouse gas emissions necessary to achieve the United States’ 2020 targets [31]. This is due to the stark difference in greenhouse gas emissions between protein sources: production of ruminant meat can have about 150 times higher global warming potential than pulse protein [32]. In addition to this significantly lower carbon footprint, a dietary shift from beef to pulses could free up 42% of cropland in the United States [31], and reduction of land used for agriculture can help prevent ecosystem damage and protect biodiversity by preventing further clearing and restoring previous cropland to open space [32]. Other studies have found differences in greenhouse gas emissions to be as much as 250-fold lower on a per gram basis in pulses than ruminant meats [33,34]. Thus, the integration of pulse consumption into climate change mitigation policy can promote sustainable development goals, and sustainable eating patterns often advocate for increasing pulse consumption. For example, the universal healthy reference diet designed by the EAT-Lancet Commission to nurture human and environmental health relies heavily on pulses and other legumes [35].

Notably, to achieve economic viability and sustainability, production should be profitable. Pulses are an economic option on farms for several reasons, including their lower requirement for external inputs, such as fertilizers. This reduces both greenhouse gas emissions and the cost of production for growers [23,36]. For instance, 35–60% less fossil energy is used on legumes than on nitrogen-fertilized cereal crops [37]. Cultivating pulses generates income in smallholder farming systems in many nations [38], and their performance in intercropping systems is especially helpful in developing countries with low-input farming systems because pulses can help increase system productivity and provide additional sources of food and income [23,24]. For example, the versatile uses by the food industry in value-added products and functional foods [39] provide growers with further markets and opportunities for profit. The diversity of varieties within each pulse crop also increases this potential because different cultivars have unique food chemistry characteristics and different types and levels of beneficial compounds [13,40,41], creating unique marketing opportunities. The rich genetic diversity that exists in pulses has not been fully utilized in breeding programs but provides tremendous potential for biofortification to benefit planetary and human health while developing additional markets [42,43]. For instance, organizations like HarvestPlus support development and distribution of iron-rich pulse varieties that can alleviate malnutrition and promote food security [42].

Unhealthy diet is one of the major risk factors for chronic disease, which is responsible for over 70% of mortalities worldwide [44]. Treatment of chronic disease is expensive, consuming two-thirds of all healthcare spending in countries like Canada and costing the United States $1.1 trillion in 2016, or about 6% of U.S. GDP [45,46]. Primary prevention is cost-effective and a sustainable approach to prevent chronic disease. Due to their numerous benefits, incorporating pulses into eating patterns actively promotes health. An analysis on the potential healthcare and societal cost savings in Canada revealed that including 100 g of pulses per day could yield up to $62.4 million in savings on annual healthcare related to type 2 diabetes and up to $315.5 million for cardiovascular disease [47]. This demonstrates the significant role pulses can play in reducing socioeconomic costs and human disease, thereby advancing sustainable healthcare systems.

Although highly important, some of these benefits for sustainability may seem intangible to consumers. Emphasizing areas of consumer interest may better motivate changes in eating patterns. For example, food waste has been the focus of popular documentaries like *Wasted! The Story of Food Waste*. Pulses can help alleviate food waste because they are shelf-stable, can be stored well in their dry or canned state for long periods of time at room temperature without losing nutritional value, and prevent leftover waste because they freeze well after being cooked. This mitigation of food waste can help eradicate malnutrition and improve food security. Moreover, pulses are nutrient-dense and an affordable source of protein, which makes them more accessible than higher cost foods of animal origin.

To motivate increased cultivation and consumption, the unique ability of pulses to promote sustainable development on an environmental, social, and economic level should be showcased. Pulses are not a “poor person’s meat”. Rather, they are the food choice of wise, conscientious global consumers who want to master cooking with a food that supports not only their own health, but also that of the planet and others. Pulses provide a novel solution to capitalize on the tight link between environmental and human health.

### 2.3. Healthfulness

Pulses are associated with a broad list of positive human health outcomes that can provide consumers a purpose to motivate consumption, such as healthy weight maintenance, gut health, and a reduced risk for cardiovascular disease and some cancers [48,49,50]. Many of the health benefits of pulses are associated with their high fiber content. Shockingly, over 90% of Americans do not consume adequate dietary fiber [51]. Although consumers are likely to have been told to eat whole cereal grains to increase fiber intake, pulses are often not mentioned despite the fact that they are two to three times richer in fiber than other grains [52]. The versatile uses of pulses will allow consumers to easily fill this fiber gap as they adopt more pulse-centric eating patterns.

Nutritional value can serve as a motivator for consumption [6], and pulses are a rich source of protein, fiber, and micronutrients like folate and iron. Making sure consumers are aware of the many health benefits of pulses is imperative. Indeed, pulses meet a wide variety of consumer demands related to health concerns, including being a rich source of plant-based protein, gluten-free, low in fat, cholesterol-free, having a low glycemic index, and fitting perfectly into diverse eating patterns, including omnivore, vegetarian, vegan, and plant-based [49]. Additionally, increasing public awareness can be achieved by incorporating information about health benefits into engaging and interactive outreach, such as hands-on classes, recipes, and materials about pulses. Hands-on experiences with recipes will also help foster the development of behavioral skills to make regular pulse consumption more feasible.

Pulses can promote short- and long-term personal and public health goals, such as weight loss, gut health, and chronic disease prevention. For example, in a recent meta-analysis by Kim and colleagues (2016), inclusion of pulses in the diet was associated with modest weight-loss even in diets that were not designed to be calorically restrictive [53]. This finding holds great potential for maintaining a healthy weight long-term. Without having to limit caloric intake, pulses may allow for weight loss or the ever-challenging maintenance of weight loss after dieting. Additionally, a high-fiber diet rich in beans has been found to be as effective as a low-carbohydrate diet for weight loss [54]. Thus, incorporating pulses may allow the public to enjoy health benefits even without restricting calories or carbohydrates, which many find undesirable and challenging. Moreover, eating pulses can help manage health concerns that are increasingly prevalent globally, such as diabetes [55,56]. These and the other numerous health benefits offered by pulses make them adaptable to diverse areas of consumer interest. To better garner public attention, outreach efforts can cater to such points, including the prebiotic effects that the fibers in pulses appear to have on gut health [57] and the fundamental role of pulses in eating patterns that are associated with positive health outcomes, such as the Mediterranean and Dietary Approaches to Stop Hypertension diets [33,58].

Although some consumers may have concerns about eating pulses, these can be readily addressed. Dispelling myths about anti-nutrients and explaining their potential benefits will be a key part of encouraging public pulse consumption. Proper preparation eliminates or significantly reduces the anti-nutrients found in pulses; for instance, heat-labile lectins are not a concern in cooked pulses [57]. This again demonstrates the importance of behavioral skills when cooking pulses. Furthermore, although anti-nutrients can reduce bioavailability of some healthful nutrients (e.g., iron), small amounts of anti-nutrients have also been linked with beneficial health outcomes, like antioxidant effects [2,49]. Flatulence is another potential barrier. However, studies suggest this concern is overexaggerated, as many individuals do not experience increased gas production, and those who do often quickly adapt to the high-fiber diet and symptoms subside [59], allowing them to reap the subsequent health and economic benefits of pulse consumption for the rest of their lives. Proper cooking of pulses, such as soaking them prior to preparation and ensuring they are fully cooked, may improve tolerability. Although annual per capita consumption of beans in the U.S. is only about 3 kg, some populations in parts of Asia and Africa have been cited as consuming over 50 kg of pulses per capita, suggesting much higher intake levels can readily be tolerated [59]. Individuals can experiment with different varieties and increasing intake slowly. Winham and Hutchins (2011) describe that when consuming ½-cup pulses per day, approximately 50% of participants reported increased flatulence from pinto beans, whereas only 19% of those eating black-eyes peas experienced perceived higher flatulence [59]. However, by the second week, the percentage of participants consuming pinto beans who perceived an increase in flatulence dropped to only 6%.

It is important to recognize that despite general awareness of health benefits; knowledge alone has proven insufficient at motivating the general public to eat more pulses. A multi-pronged approach that emphasizes health benefits of personal interest (i.e., explains the benefits of pulses against diabetes to a person with diabetes) and provides more details and clarity about health statements may help remedy this. A study by Farrell and colleagues (2019) found that when participants perceived a health claim on a bag of beans to have personal relevance, they were more likely to report higher intentions for increased bean consumption [60]. These same participants also wanted quantification of benefits, with details on the amount to eat and how much of a benefit they could expect (i.e., how many grams should they eat to lower cholesterol, and how much of a reduction could be expected) [60]. Thus, instead of a generic statement that pulses are healthy, it could be more beneficial to provide details about specific health outcomes desired by consumers. Furthermore, although it will require more research, defining a recommended serving size of pulses for maximal health benefits would provide more clarity and direction [61], removing the ambiguity of vague statements to simply ‘eat more pulses’. Specific and actionable tips can empower consumers and foster the development of new behaviors, such as including recipe cards with sampling at grocery stores on three quick, tasty, and easy ways to start including ½-cup of pulses at breakfast.

## 3. Synthesis and Critical Analysis

The Food and Agriculture Organization declared 2016 the International Year of Pulses. Despite such efforts, pulse consumption remains low in many countries. However, consumer interest in healthful, environmentally friendly food is constantly evolving. Simultaneously, consumers want food that is affordable, convenient, and delicious [20]. Pulses are uniquely positioned to meet all these demands and be a staple food in diverse eating patterns. Additionally, they store well for long periods of time, a factor that has become increasingly desirable during the COVID-19 pandemic.

**Figure 2:**
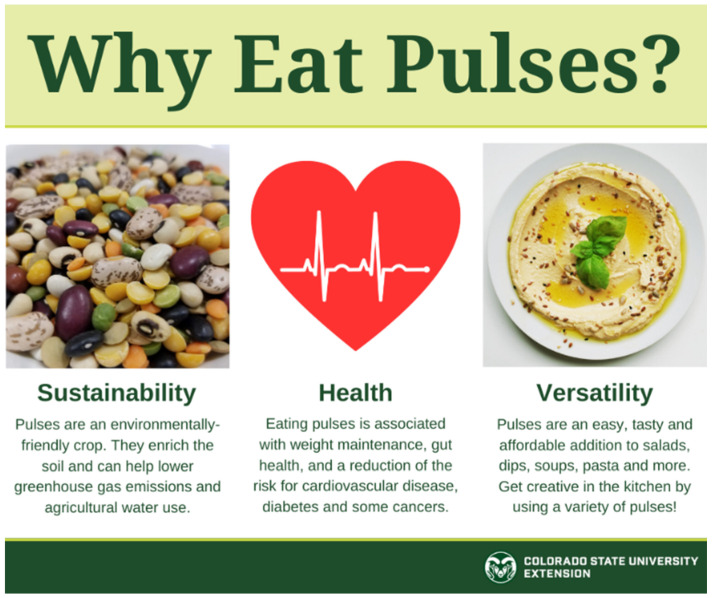
An example of pulse-centered outreach through Extension’s social media network, promoting three important aspects of pulses: sustainability, health, and versatility.

It is important to provide the public with information about pulses that will help address perceived barriers to consumption, such as the false perception that increased flatulence is inevitable [59]. However, knowledge alone about the human health and sustainability benefits of pulses appears to have been insufficient to change public eating patterns. The COVID-19 pandemic resulted in increased consumer purchases of pulses, but to truly change eating habits long-term, motivation and behavioral skills are also critical [9]. The food media, restaurant industry, and organizations like USDA, USA Dry Pea & Lentil Council, the American Pulse Association, and Extension will play a vital role in fostering behavioral skills that allow for increased consumption, such as preparation tips and recipes to regularly include pulses during any meal or snack time. As pulses are increasingly highlighted through such outlets (see Figure 2 for a sample social media post by Extension), consumption will become more of a social norm, a key aspect of social motivation.

In addition to the social factor, motivation on a personal level is also critical [9]. On its own, background knowledge about the health and environmental benefits of pulses may not be enough to influence attitudes and beliefs, which are essential for personal motivation. However, if information and behavioral skills are coupled with regular exposure to pulses (i.e., through media, government, and other outlets) and an individual can personally experience some of the benefits of pulses (e.g., affordability, convenience, taste, and health benefits like weight maintenance), this could change consumer attitudes. Furthermore, consumers can be further motivated as they: (1) relish autonomy and creativity in the kitchen with the versatility offered by pulses; (2) increasingly master the use of pulses in their eating patterns and enjoy the results; and (3) recognize the purpose of eating pulses—to benefit personal, public, and planetary well-being.

Despite the long list of positive outcomes of pulses on human and environmental health, culinary versatility may be one of the most appealing aspects of pulses to emphasize when motivating the average consumer. Unlike other crops that are more limited in application, pulses can be deliciously prepared as whole foods, in mixed dishes, and in other food products at an economical price point. Furthermore, there is a rich global history of pulse-based recipes. Combined with current media coverage and the renewal of consumer interest in and purchases of pulses, now is the time to motivate people to change their eating habits to regularly enjoy this highly versatile food.

Governments, policymakers, researchers, public health workers, and local institutions also have a role to play by creating policies and environments that encourage production by growers, allow consumers regular access to pulses, and provide relatable, actionable tips on how to include them in meals. For more holistic and successful changes, it can be beneficial to combine elements of both top-down (i.e., infrastructural and policy changes made by a few individuals) and bottom-up approaches (i.e., involving the public in the decision- and change-making processes) [62]. Top-down efforts can centralize communication and ensure key policymakers are onboard, and bottom-up approaches ensure community buy-in [63]. Such efforts will be key to promoting pulses as a staple food of wise, conscientious consumers and make adoption of pulse-centric earing patterns approachable and attainable (e.g., increase consumption a ½-cup at a time).

## 4. Final Comment

Will we continue to knowingly ignore an achievable solution to critical global health challenges we face today? Indeed, pulses are a poster child for showing that although knowledge is important, knowledge alone does not result in behavioral change. Providing multiple widely documented benefits for human and environmental health at an economical price, eating more pulses may seem like a commonsense decision. Yet, pulses are dramatically under consumed, with precipitous declines in the diet inversely related to rises in chronic diseases. Purchases increased during the COVID-19 pandemic, demonstrating that consumers recognize some of the advantages of pulses. Now, it is time to seize this opportunity and ensure that this trend continues. Continuing research into the benefits of pulses is indispensable to support the dissemination of knowledge pertinent to consumers and policymakers alike. However, to realize public adoption of pulse-centric eating patterns, we must simultaneously fuel consumer motivation. Engaging outreach should emphasize the culinary versatility of pulses and the ability to seamlessly include them in meals and snacks. As consumers further their sense of autonomy and mastery through exploration of pulses in the kitchen, they will also be motivated by the sense of purpose they experience from contributing to sustainability at personal, economic, and planetary levels. When promoting the positive health effects of pulses, it is important to capitalize on areas of consumer interest and provide more concrete health statements and recommendations that are quantified when possible. Ultimately, capitalizing on all that pulses have to offer allows for a delicious eating pattern that simultaneously advances food security, human health, and environmental sustainability.
